# A systematic review and meta-analysis of circulating serum and plasma microRNAs in TB diagnosis

**DOI:** 10.1186/s12879-024-09232-0

**Published:** 2024-04-15

**Authors:** Harinisri Gunasekaran, Pavithra Sampath, Kannan Thiruvengadam, Muniyandi Malaisamy, Rathinasabapati Ramasamy, Uma Devi Ranganathan, Ramalingam Bethunaickan

**Affiliations:** 1 Department of Immunology, ICMR-National Institute for Research in Tuberculosis, No.1. Mayor Sathyamoorthy Road, 600 031 Chetpet, Chennai, India; 2 Department of Epidemiology Statistics, ICMR-National Institute for Research in Tuberculosis, Chennai, India; 3 Department of Health Economics, ICMR-National Institute for Research in Tuberculosis, Chennai, India; 4 Library and Information Center, ICMR-National Institute for Research in Tuberculosis, Chennai, India; 5 University of Madras, Chennai, India

**Keywords:** Tuberculosis, microRNA, Biomarker, miR-197, miR-144, TB diagnosis

## Abstract

**Background:**

Tuberculosis (TB) ranks as the second leading cause of death globally among all infectious diseases. This problem is likely due to the lack of biomarkers to differentiate the heterogeneous spectrum of infection. Therefore, the first step in solving this problem is to identify biomarkers to distinguish the different disease states of an individual and treat them accordingly. Circulating microRNA (miRNA) biomarkers are promising candidates for various diseases. In fact, we are yet to conceptualize how miRNA expression influences and predicts TB disease outcomes. Thus, this systematic review and meta-analysis aimed to assess the diagnostic efficacy of circulating miRNAs in Latent TB (LTB) and Active Pulmonary TB (PTB).

**Methods:**

Literature published between 2012 and 2021 was retrieved from PubMed, Web of Science, Cochrane, Scopus, Embase, and Google Scholar. Articles were screened based on inclusion and exclusion criteria, and their quality was assessed using the QUADAS-2 tool. Funnel plots and forest plots were generated to assess the likelihood of study bias and heterogeneity, respectively.

**Results:**

After the screening process, seven articles were selected for qualitative analysis. The study groups, which consisted of Healthy Control (HC) vs. TB and LTB vs. TB, exhibited an overall sensitivity of 81.9% (95% CI: 74.2, 87.7) and specificity of 68.3% (95% CI: 57.8, 77.2), respectively. However, our meta-analysis results highlighted two potentially valuable miRNA candidates, miR-197 and miR-144, for discriminating TB from HC. The miRNA signature model (miR197-3p, miR-let-7e-5p, and miR-223-3p) has also been shown to diagnose DR-TB with a sensitivity of 100%, but with a compromised specificity of only 75%.

**Conclusion:**

miRNA biomarkers show a promising future for TB diagnostics. Further multicentre studies without biases are required to identify clinically valid biomarkers for different states of the TB disease spectrum.

**Systematic review registration:**

PROSPERO (CRD42022302729).

**Supplementary Information:**

The online version contains supplementary material available at 10.1186/s12879-024-09232-0.

## Background

TB caused by the airborne pathogen *Mycobacterium tuberculosis* (Mtb) is a major health challenge in many countries owing to its complex immunological response, chronic progressive nature, and emergence of drug resistance [1, 2]. The most recent Global TB Report (2022) by the World Health Organization (WHO) reported TB as the second leading cause of death from a single infectious agent with 1.4 million deaths in 2021 [3] whereas it was the 13th leading cause of death in 2019 [4]. Clearly, current data imply the need for more robust tools to curtail this disease.

TB is a heterogeneous spectrum of infection rather than an oversimplified binary classification of TB into active TB and LTB, which only represents the extremes of the spectrum [5]. LTB is an umbrella term that includes many asymptomatic stages, such as latency and subclinical or incipient infections. The different stages of the disease can be evidenced through blood or sputum tests by observing the changes in the biomarkers; however, data from previous studies have shown that these markers remain elusive. Thus, the global goal to end TB is severely impeded by the lower efficacy of the current TB diagnostic tools, their inability to identify drug resistance, and their inability to be applied as point-of-care testing [6]. The most commonly used gold standard tests are sputum culture, which involves isolation of Mtb in culture; however, Mtb usually takes a longer time to grow, and Xpert MTB/RIF, which helps in the early detection of Mtb and simultaneously assesses resistance to rifampicin (RIF). Although the latter is far better than the conventional sputum smear, this assay is expensive [7, 8]. Additionally, conventional testing methods cannot predict disease progression. Hence, one of the high-priority research areas in TB elimination is the identification of biomarkers to rapidly detect TB and differentiate it from other subclinical conditions in the TB disease spectrum.

miRNAs are single-stranded, small, non-coding RNA molecules that play regulatory roles in many biological processes [9] and perform post-transcriptional modifications of protein-coding genes [10]. These play important roles in a multitude of developmental and physiological processes, such as cell cycle control, hematopoiesis, apoptosis, and neurological development [11]. Since their discovery, numerous miRNAs have been reported to be either found intracellularly or secreted into the extracellular fluid directly, or in the form of exosomal vesicles. A review of circulating biomarkers has shown the diagnostic potential of different miRNAs associated with various diseases, such as TB, sepsis, hepatitis, and pertussis [12]. The high stability of serum circulating miRNAs makes them potential non-invasive biomarker candidates for the early detection of diseases rather than protein biomarkers, which have lower sensitivity and specificity [13, 14]. However, there are several challenges for a miRNA biomarker to enter clinical practice because of the variability and irreproducibility between studies [15]. Circulating miRNAs perform multiple functions. They have multiple targets and exogenous miRNAs may display off-target effects [16]. Their expression sometimes overlaps with other comorbidities, which explains why it is a challenge for miRNA biomarkers to enter clinical practice, and this must be resolved to build a successful miRNA biomarker. To enhance their therapeutic effects, miRNA expression can be modulated by various mechanisms such as positive or negative regulation, miRNA mimics, and miRNA replacement therapy [16]. Even though the human host has developed several strategies like phagocytosis, apoptosis and autophagy to overcome the invading pathogen, in the case of TB, Mtb has evolved several mechanisms to evade the host immune defences [17]. One such mechanism involves hijacking and manipulating host miRNAs for intracellular survival. Interestingly, many valuable studies have shown changes in miRNA expression profiles mediated by Mtb infection, with unique patterns of upregulation and downregulation depending on the strain, virulence, live status, and host immunity [18]. Mtb requires a lipid-rich environment, for which it relies on the host cell by altering the host microenvironment [19]. For example, miR-155, which plays a regulatory role in cholesterol uptake, is overexpressed in macrophages infected with Mtb [20], and another study has shown that its upregulation targets the transcription factor forkhead box O3 (FOXO3), thereby inhibiting macrophage apoptosis [19, 21]. Another successful way to establish infection is by eliminating autophagy, which is critical for host immune defense. Mtb downregulates miR-25 and inhibits autophagy by preventing the fusion of autophagosomes and lysosomes [19, 22]. These data strongly suggest that circulating miRNAs could be considered promising blood-based biomarkers for detecting different stages of the TB disease spectrum. However, there is still a lack of meta-analysis data on the role of circulating miRNAs as diagnostic markers for the TB disease spectrum.

Hence, in this study, considering the significance of miRNAs, we performed a systematic review and meta-analysis of all eligible studies to determine the diagnostic accuracy of potential miRNAs as circulating biomarkers and their role in TB pathogenesis.

This systematic review has been registered in PROSPERO (CRD42022302729).

## Methods

### Search Strategy

We conducted this systematic review and meta-analysis in accordance with the Preferred Reporting Items for Systematic Reviews and Meta-Analyses (PRISMA) guidelines [23]. An extensive literature search was carried out using search engines (PubMed, Web of Science, Cochrane, Scopus, Embase, and Google Scholar) to retrieve all articles related to circulating miRNAs in TB. Specific search strategies, including MeSH terms and keywords applied for the search, are attached to Additional File 1: Table S1.

### Eligibility criteria

#### Inclusion criteria

Articles were selected based on the following criteria: studies reporting the expression of individual or panel of circulating (plasma or serum) miRNAs in TB. When a study included both the miRNA assay and other tests, only miRNA data were extracted. Adequate data should be available for effective evaluation of the diagnostic performance of these circulating miRNAs. All study designs (cross-sectional, case-control, and cohort studies) were considered for analysis, without regard to prospective or retrospective sample collection. All the relevant articles published in English between January 2012 and December 2021 were included in this study.

#### Exclusion criteria

Studies were excluded if they met the following criteria: studies evaluating miRNAs as a biomarker for diseases other than TB; studies lacking sensitivity, specificity, and area under the curve (AUC) data; studies without full text or with incomplete data, narratives, letters, editorials, systematic reviews, meta-analyses, conference abstracts, repeated publications, animal studies, and studies conducted on the paediatric population.

### Screening and data extraction

The study articles retrieved from the database search were initially screened for titles and abstracts to remove duplicates and identify potentially eligible studies. Two reviewers independently screened the articles based on the eligibility criteria to filter out relevant studies. Data such as the author’s first name, publication year, country, sample size (cases and controls separately), male/female population, mean or median age, study population (PTB/LTB/HC), index test, reference test, source of sample, screening and validation methods, individual miRNA/miRNA signatures identified, and diagnostic accuracy measures such as sensitivity, specificity, AUC, True Positives (TP), False Positives (FP), True Negatives (TN), and False Negatives (FN) were extracted. In the absence of these data, the reported sensitivity, specificity, and sample size were used to determine TP, FP, TN, and FN values. Based on this, a data table was generated and entered in MS Excel.

### Quality assessment of the included studies

The Quality Assessment of Diagnostic Accuracy Studies-2 (QUADAS-2) tool was used to assess the quality of the included studies. This tool includes four domains: patient selection, index test, reference standard, and flow and timing. These domains consist of questions used to assess the risk of bias and clinical applicability. The risk of bias level can be determined as “low,” “high” or “unclear” based on the answers to the questions of each domain.

### Statistical analysis

First, we summarized the study characteristics using the frequency, percentage, mean, and standard deviation. To assess the heterogeneity, we examined the data using a forest plot. Subsequently, we calculated the between-study variance (heterogeneity) and standard deviation using the DerSimonian-Laird estimator and Jackson method, respectively, to determine the 95% Confidence Intervals (CI) with adjustments. Furthermore, we estimated the I^2^ statistic (with 95% CIs), which represents the ratio of the observed heterogeneity to the total observed variance. Finally, we conducted a formal χ2 test with Cochran’s Q statistic to assess the common effect size across all studies. To identify outliers and influential studies contributing to heterogeneity, we used a diagnostic Baujat plot. Additionally, we performed a series of leave-one-out diagnostic tests to calculate pooled estimates by excluding one study at a time from the analysis.

To compute the pooled sensitivity and specificity of miRNAs, we used a random effects model by assigning weights to each study based on the inverse of the total variance. Furthermore, we conducted subgroup analysis to evaluate the performance of miRNAs in the context of HC vs. TB and LTB vs. TB. All statistical tests were two-sided with a fixed p-value of 0.05. All analyses were conducted using R software version 4.1.1 (R Core Team, 2020). We utilized the “tidyverse”, “metafor” and “meta” packages.

## Results

### Literature retrieval and screening

**Fig. 1:**
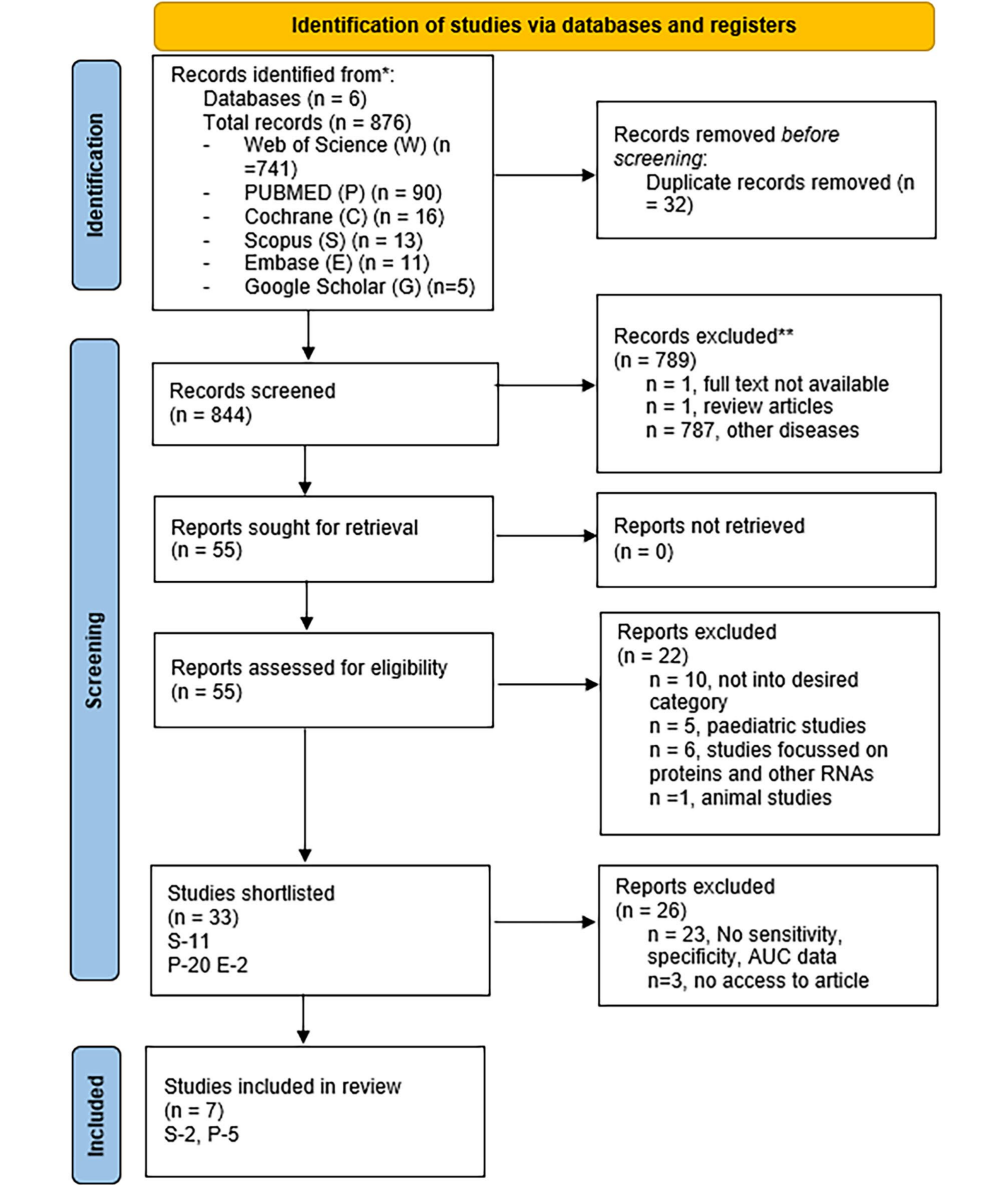
PRISMA flowchart

We obtained 876 articles, of which 32 duplicates were excluded. Of the remaining 844 articles, 789 were excluded after filtering the titles and abstracts. Of the 55 remaining articles examined, 22 were excluded for the following reasons: not in the desired category (*n* = 10), pediatric studies (*n* = 5), studies focusing on proteins and other RNAs (*n* = 6), and animal studies (*n* = 1). Finally, from the remaining 33 articles, seven studies were selected for our meta-analysis after excluding studies that did not report the sensitivity, specificity, or AUC value of significant miRNAs [24–30]. The PRISMA flowchart describing the screening and selection criteria is shown in Fig. 1.

The flow diagram of the study selection detailing the databases searched, number of articles shortlisted and the studies included in review.

### Characteristics of included articles

The characteristics of the seven studies are tabulated in Table-1. The articles included were published between 2013 and 2021. All studies focused on biomarkers for the diagnosis of PTB, including both drug-sensitive TB (DS-TB) and drug-resistant TB (DR-TB) groups and differentiation from HC, and only one study on latently infected individuals. The sample size of the selected studies ranged from 24 to 124 in the PTB group, 35 in the LTB group, and 15 to 117 in the HC group, with people belonging to multiple ethnicities. Four out of seven studies used serum, two used plasma, and one used serum exosomes. miRNA screening was based on miRNA databases, RNA sequencing, and microarrays. Significant miRNAs were validated using quantitative real-time polymerase chain reaction (qRT-PCR) in all studies. Subgroup analysis was performed for HC vs. TB and LTB vs. TB. A total of 13 individual miRNAs and four miRNA signatures were identified from the seven selected studies and labelled A to W for sensitivity and specificity assays (Table 1).

**Table 1:** Basic characteristics of included studies

Authors	Year	Country	Sample used	Patient Characteristics						Diagnosis	Screening method	Validation method	Biomarker utility	miRNA profile	Labeling for RF plot	Group definition for RF plot
				Control	Male/Female	Age	Cases	Male/Female	Age							
Abd-El-Fattah et al.,	2013	Mexico	Serum	37 HC	21/16	50.1 ± 14.2	29 PTB	17/12	47.7 ± 9.8	TST		qRT-PCR	HC vs. PTB	miR-197↑	A	HC vs. TB
Carranza C et al.,	2021	North Western China	Serum (Exosomes)	15 HC	10/5	45 (27–61)	24 DR-TB	18/11	43.5 (19–77)	PTB: Confirmed diagnosis of TB and were under Directly Observed Therapy	TaqMan RT-qPCR	TaqMan Advanced miRNA assays	HC vs. DR-TB	miR-197-3p↓	B	HC vs. TB
														miR-223-3p↓	C	HC vs. TB
														miR-let7e-5p↑	D	HC vs. TB
														miRs(197-3p + let-7e-5p +223-3p) (Differential expression)	E	HC vs. TB
													HC vs. MDR-TB	miR-197-3p↓	F	HC vs. TB
														miR-223-3p ↓	G	HC vs. TB
														miR-let7e-5p↑	H	HC vs. TB
														miRs(197-3p + let-7e-5p +223-3p) (Differential expression)	I	HC vs. TB
EN Ndzi et al.,	2019	Cameroon	Plasma	43 HC	13/29	27 ± 7	84 PTB 35 LTB	PTB: 57/26 LTB: 8/27	PTB: 33 ± 12 LTB: 34 ± 11	PTB: Microscopy or culture (0, 2, 6) LTB: Positive QFT HC: Negative QFT	Database reported miRNAs	miRCURY LNA SYBR Green PCR kit (Qiagen, Maryland, USA)	HC vs. PTB	hsa-miR-29a-3p↑	J	HC vs. TB
														hsa-miR-361-5p↑	K	HC vs. TB
													LTB vs. PTB	hsa-miR-29a-3p↑	L	LTB vs. TB
														hsa-miR-361-5p↑	M	LTB vs. TB
Huihui Tu et al.,	2019	China	Serum	86 HC	50/36	21-64	108 PTB	66/42	18-72	PTB: Diagnostic criteria of the Ministry of Health, China	Solexa sequencing	SYBR green qRT-PCR	HC vs. PTB	miR-17- 5p, miR-20b-5p, miR-423-5p↑	N	HC vs. TB
SE Barry et al.,	2018	Egypt	Plasma	100 HC	57/43	35 (18-78)	100 PTB	58/42	43 (19-91)	PTB: Clinical and radiographic findings and sputum smear microscopy and mycobacterial culture HC: Interview and chest radiography	Plasma Focus miRNA PCR Panels (Exiqon)	qRT-PCR (Exiqon)	HC vs. PTB	miR-29a, miR-99b, miR-21, miR-146a, miR-652 (Differential expression)	O	HC vs. TB
Yan Lv et al.,	2015	China	Serum	117 HC	70/47	27.49 ± 8.80	124 PTB	70/54	27.25 ± 8.99	PTB: Typical TB clinical symptoms, bacterial culture, and imaging examinations		qRT-PCR	HC vs. PTB	miR-144↑	P	HC vs. TB
Xing Zhang et al.	2013	China	Serum	88 HC	65/23	48 (24–67)	108 PTB	73/35	45 (14–62)	PTB: clinical manifestations, bacterial culture, and radiographic findings		SYBR green qRT-PCR assay	HC vs. PTB	hsa-miR-378↓	Q	HC vs. TB
														hsa-miR-483-5p↓	R	HC vs. TB
														hsa-miR-22↓	S	HC vs. TB
														hsa-miR-29c↓	T	HC vs. TB
														hsa-miR-101↑	U	HC vs. TB
														hsa-miR-320b↑	V	HC vs. TB
														hsa-miR-378, hsa-miR-483-5p, hsa-miR-22, hsa-miR-29c, hsa-miR-101, hsa-miR-320b (Differential expression)	W	HC vs. TB

### Qualitative assessment of the included studies

**Fig. 2:**
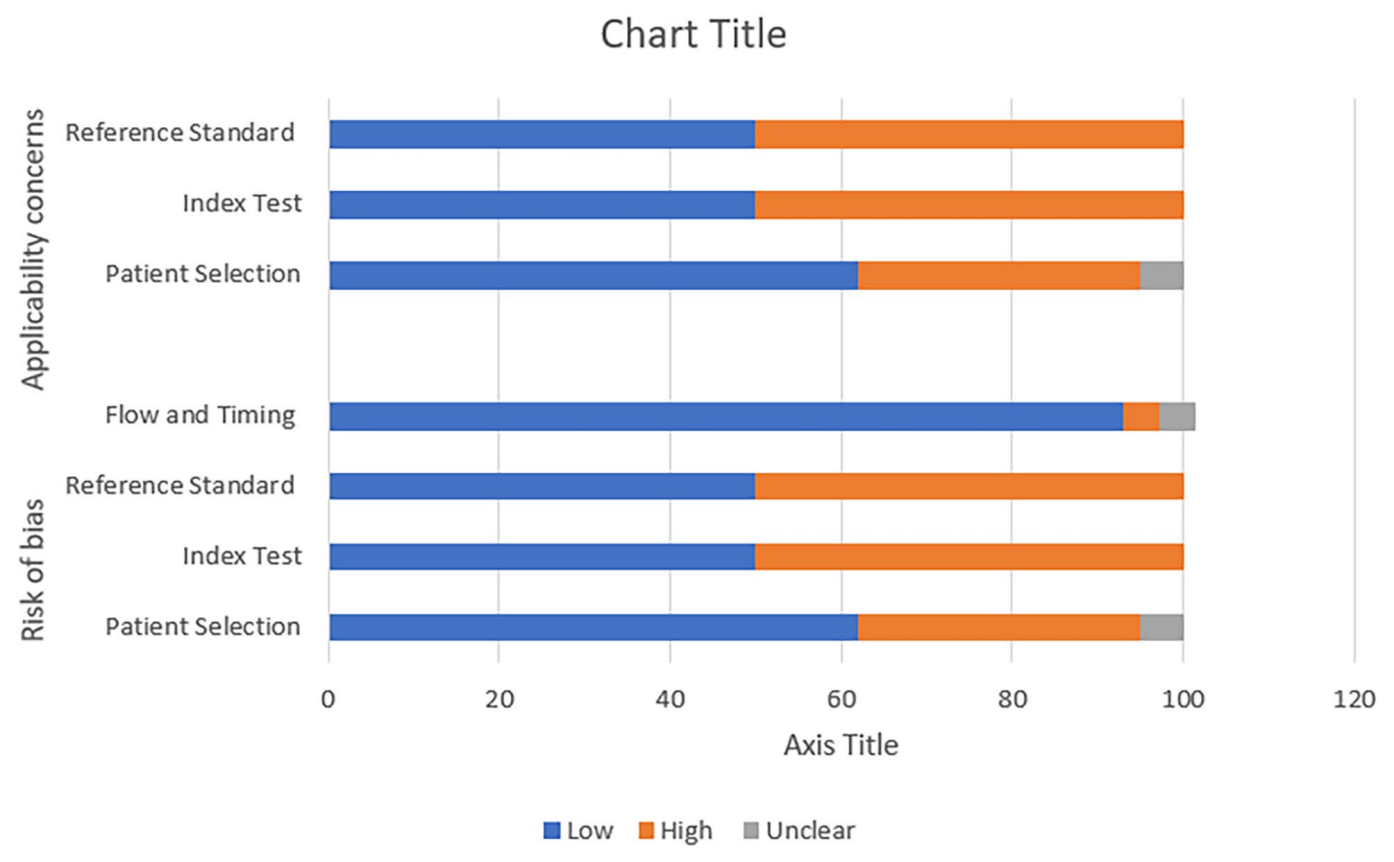
Risk of bias and applicability-concerns graph presenting authors’ judgments based on the QUADAS-2 tool

The results of the quality assessment using the QUADAS-2 are shown in Fig. 2. The included studies had a low risk of bias (< 50%). Applicability concerns were also very low in all domains. The evaluation results indicated that the overall quality of the articles was high.

Based on the assessment, the risk of bias was found in the selected studies due to patient selection issues, inadequate reference standards, index tests and flow and timing issues. The applicability concerns were also found to be high in the studies.

### Quantitative assessment of the included studies

The meta-analysis involved statistical analysis of the heterogeneity of the reported circulating miRNAs that differentiate PTB from HC or LTB. Given that the studies were conducted in different regions with varying characteristics and a limited number of studies, heterogeneity was expected. To evaluate potential bias in the studies, we created a funnel plot. The data points on the funnel diagram displayed an uneven distribution, and the heterogeneity test also indicated statistical significance (*p* < 0.05), suggesting the likely presence of publication bias (Additional File 1: Figure S1 and S2). The Baujat plot demonstrated the contribution of each study to the overall Q-test statistic for heterogeneity compared with their influence on the overall estimate based on an equal-effects model, both with and without the study included in the model fitting (Additional File 1: Figure S3). This plot reveals that the studies by Yan et al. [27] and Barry et al. [25] had a greater impact on the pooled estimates. However, we did not exclude any studies, as there was no significant reduction in heterogeneity, as demonstrated in the leave-one-out diagnostic tests (Additional File 1: Figure S4). This bias may be attributed to the inconsistent reporting of miRNAs between the PTB and LTBI groups, as well as the small sample size. Consequently, our meta-analysis included only miRNAs that were consistently reported across different group comparisons, ensuring the reliability of utilizing circulating miRNAs for diagnosing PTB and LTBI.

### Circulating miRNAs as biomarkers of TB diagnosis

**Fig. 3:**
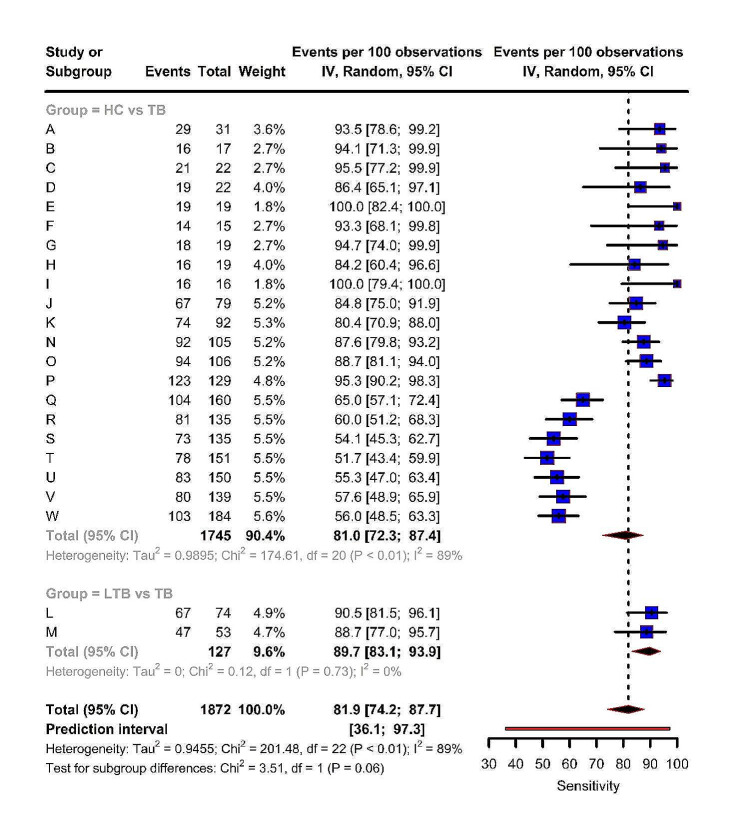
Forest plot representing pooled and miRNA-wise sensitivity estimates from the included studies

Overall, the sensitivity and specificity of the pooled estimates, as well as the individual estimates from each study, were summarized using forest plots. The individual miRNAs, miR-144 and miR-197 showed higher sensitivity and specificity in discriminating TB from HC. The overall pooled sensitivity was estimated to be 81.9% (95% CI: 74.2, 87.7), while the estimates for HC vs. TB and LTB vs. TB were 81.0% (95% CI: 72.3, 87.4) and 89.7% (95% CI: 83.1, 93.9) respectively. The model provided a wide prediction interval of 36.1– 97.3%, indicating a high level of heterogeneity (τ^2^ = 0.946, I^2^ = 89.0%, p-value < 0.01). There was a slight difference in heterogeneity between the sub-groups, with the two miRNAs in the LTB vs. TB group showing more similarity (*p* = 0.06) (Fig. 3).

The figure shows the sensitivity of different miRNAs in all included studies. Visual inspection of the forest plot indicates considerable heterogeneity in sensitivity estimates.

**Fig. 4:**
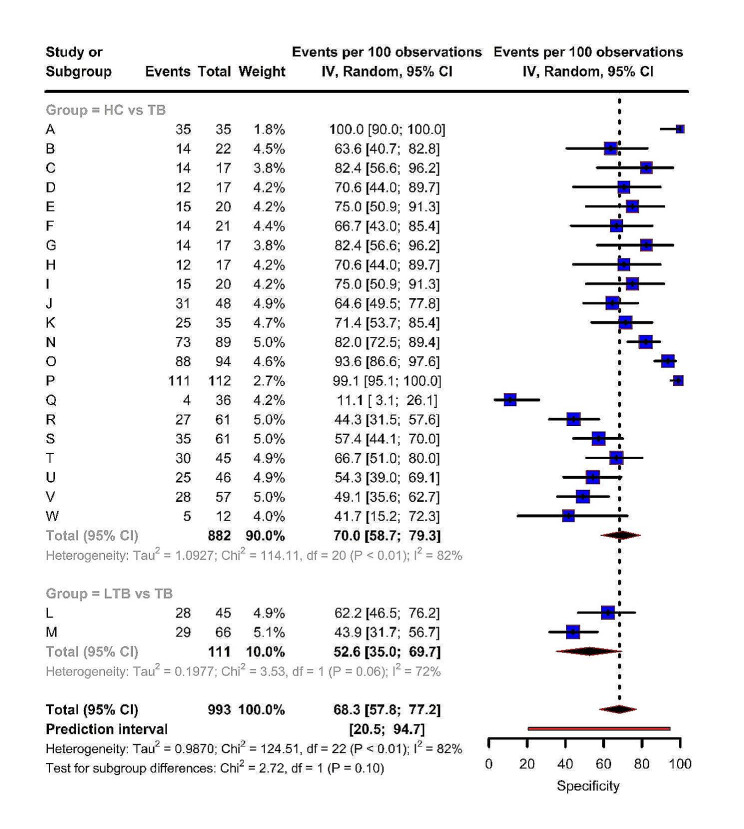
Forest plot representing pooled and miRNA-wise specificity estimates from the included studies

Similarly, the overall pooled specificity was estimated to be 68.3% (95% CI: 57.8, 77.2), while the estimates for HC vs. TB and LTB vs. TB were 70.0% (95% CI: 58.7, 79.3) and 52.6% (95% CI: 35.0, 69.7) respectively. The model provided a wide prediction interval of 20.5– 94.7%, indicating a high level of heterogeneity (τ^2^ = 0.987, I^2^ = 82.0%, p-value < 0.01). There was no significant difference in heterogeneity between the sub-groups (*p* = 0.10) (Fig. 4).

The figure shows the specificity of different miRNAs in all included studies. Visual inspection of the forest plot indicates considerable heterogeneity in specificity estimates.

From the extensive analysis, we conclude that miR-197 (sensitivity:94%, specificity:100%) and miR-144 (sensitivity:95%, specificity:99%) had good diagnostic abilities to diagnose TB from HC and the miRNA signature (miR197-3p, miR-let-7e-5p and miR-223-3p) had the ability to diagnose DR-TB with a sensitivity of 100% but with a specificity of only 75%.

## Discussion

TB is characterized by the presence of chronic inflammatory granulomatous lesions in the lung, which mark the persistent state of infection formed by the complex interaction between the host and the bacterium [31]. This granuloma contains the bacteria thereby preventing their spread and establishing latency, but with a decline in immunity, this protective structure disrupts and spreads the bacteria leading to lung damage and disease [32]. Almost 1/4th of the world’s population is latently infected and it is difficult to treat all of them, making early prediction the most probable solution for prevention.

Currently, no diagnostic tool can predict disease progression or discriminate between TB and LTB. This lack of diagnostic tools severely hampers TB eradication and therefore the current research focuses on identifying new potential biomarkers other than the conventional testing methods which could greatly help reduce the TB burden. Recently, liquid biopsy biomarkers such as miRNA have drawn attention as prognostic biomarkers owing to their stability and unique expression patterns in different disease states [33]. Researchers have invested time in accumulating evidence regarding these miRNAs and their function in TB through review articles [34], meta-analyses and systematic reviews [35, 36]. However, the knowledge on the complete profile of circulating miRNAs is limited. To address this, we performed a systematic review and meta-analysis restricting to circulating miRNAs and their expression in TB and identified miRNAs with biomarker abilities of good specificity and sensitivity that can detect TB. Our preliminary findings were as follows: (1) miR-197 (sensitivity:94%, specificity:100%) and miR-144 (sensitivity:95%, specificity:99%) had good diagnostic ability to diagnose TB from HC with higher sensitivity and specificity values and (2) the miRNA signature model (miR197-3p, miR-let-7e-5p and miR-223-3p) could diagnose DR-TB with a sensitivity of 100% but with a specificity of only 75%.

Since the circulating extracellular miRNAs can easily be isolated and are readily quantifiable using RT-PCR, they are suitable biomarker candidates to quickly identify infectious diseases [37]. miRNA based therapeutics is an emerging field and several miRNA molecules are currently in different phases of clinical trials. Miravirsen, (antisense to a human miRNA miR-122) which targets miR-122, the first drug to enter clinical trials, is under phase II clinical trial for the treatment of Hepatitis C virus (HCV) infection [38]. The regulatory effects and differential expression of miRNAs in TB have been well studied and various signatures have been found to be promising candidates for diagnosing TB and LTB [10, 39]. Each miRNA studied in this meta-analysis was found to have a unique predictive effect on TB. Among them, the upregulation of miR-197 and miR-144 in TB compared to HC was found to be highly significant with better diagnostic ability. miR-197-3p is a neutrophil associated miRNA which inhibits the protective cytokine, IL-22 and their downregulation increases the chance of protection to TB [40]. In contrast, miR-197 was significantly upregulated in the selected studies, suggesting its role in disease burden and severity in both DS and DR-TB. In addition, the combination of miR-197-3p, miR-let-7e-5p, and miR-223-3p has been proposed for the efficient diagnosis of DR-TB [26]. Lyu et al. found that miR-let-7e-5p expression was specific to the LTB group [41]. In contrast, in-vitro studies have shown that its levels are upregulated following the course of the Mtb infection which inhibits host macrophage apoptosis in the infected cells by inhibiting the key caspase, CASP3 [42]. The upregulation of miR-223-3p was found to suppress Mtb induced inflammation [43] since it was found to target the STAT1 gene which plays a role in interferon-gamma (IFN-γ) signalling [44]. However, the specificity of the trio miRNA model (miR-197-3p↑ + miR-let-7e-5p↓ + miR-223-3p↑) in the meta-analysis did not meet the acceptable range for diagnosis. Whereas, miR-223-3p alone showed better sensitivity (95%) and specificity (82%) to discriminate DR-TB and MDR-TB from HC. The other promising candidate miR-144 reported in Yan Lv et al. was found to be upregulated in the TB group compared to the HC group, similar to the findings of Wang et al. [27, 39]. They are known to regulate T-cell proliferation and autophagy [45, 46], thereby playing a vital role in TB infection.

The current study finding showed that the diagnostic performance of miRNAs was better in HC vs. TB than that in the LTB vs. TB. The upregulation of two miRNAs, miR-29a-3p and miR-361-5p showed good sensitivity but poor specificity in distinguishing TB from LTB. This is due to the limited number of studies, as only one study mentioned miRNAs that could discriminate TB from LTB. The authors claimed they were the first to describe these signatures and mentioned that only miR-29a-3p had better diagnostic efficiency and miR-361-5p was not good enough [28].

The current meta-analysis data suggest two potential miRNA candidates (miR-197 and miR-144) for discriminating TB from HC. However, the overall miRNA assay showed poor diagnostic performance in TB, with a pooled sensitivity of 81.0% (95% CI: 72.3, 87.4) and specificity of 70.0% (95% CI: 58.7, 79.3) for the HC vs. TB group and 89.7% sensitivity (95% CI: 83.1, 93.9) and 53% specificity 52.6% (95% CI: 35.0, 69.7) for the LTB vs. TB group. The qualitative assessment shows a low risk of bias which implies that the selected articles have reported low bias in their findings but their data points are highly heterogeneous while estimating the pooled estimates. miRNAs are involved in a wide variety of bodily functions and a single miRNA may play a role in multiple co-morbidities. Significant differences exist among studies in miRNAs that have been identified as possible biomarkers. It is obvious that what matters is how each person’s miRNAs are expressed and it highly varies between the individual depending on their co-morbid conditions and their immune system. Thus, the field of miRNA studies has a lengthy history of producing questionable figures due to these challenges. A central reason for the lack of reproducibility and consistency among the reported miRNAs and/or miRNA signatures was mainly attributed to the heterogeneity of the reported studies and the smaller sample size. Further experimental validation and future studies are needed to review the performance of the suggested circulating miRNA candidates.

The notable strength of this systematic review was that the included studies were selected after a thorough quality assessment. However, the study has some limitations. First, in this study, we obtained only a limited number of articles (seven) which makes the data insufficient to give a strong conclusion. Second, the study population and the suggested miRNAs were inconsistent among the studies because we included studies from multiple ethnicities and across different age groups and might be due to the usage of different screening platforms. Therefore, the result may be over- or underestimated among the included studies. Finally, the funnel plot suggests the existence of publication bias; therefore, our results must be interpreted with caution. Despite the limitations of our review, it summarizes the role of circulating miRNAs in TB as a diagnostic marker and could be used as a reference for the future studies.

### Electronic supplementary material

Below is the link to the electronic supplementary material.

Supplementary Material 1

## Data Availability

The data supporting the findings of this review will be made available by the corresponding author, upon request.

## Abbreviations

AUC: Area Under the Curve
CI: Confidence Intervals
DR-TB: Drug-Resistant TB
DS-TB: Drug-Sensitive TB
HC: Healthy Control
HCV: Hepatitis C Virus
FN: False Negatives
FOXO3: Forkhead box O3
FP: False Positives
IFN-γ: Interferon-gamma
LTB: Latent TB
miRNA: microRNA
Mtb: *Mycobacterium tuberculosis*
PRISMA: Preferred Reporting Items for Systematic Reviews and Meta-Analyses
PTB: Pulmonary TB
qRT-PCR: quantitative Real-Time Polymerase Chain Reaction
QUADAS-2: Quality Assessment of Diagnostic Accuracy Studies-2
RIF: Rifampicin
TB: Tuberculosis
TN: True Negatives
TP: True Positives
WHO: World Health Organization
