# Supplementary material for: A systematic review and meta-analysis of circulating serum and plasma microRNAs in TB diagnosis
Source: BMC Infect Dis. 2024 Apr 15;24:402. doi: 10.1186/s12879-024-09232-0 (PMC11017603; doi:10.1186/s12879-024-09232-0)
Supplement: Supplementary file 1 — Supplementary Material 1 [file 12879_2024_9232_MOESM1_ESM.docx]

**Supplementary Information**

**Additional File 1:**

**Figure S1.** Sensitivity Funnel Graph of the included studies. **Figure S2**. Specificity Funnel Graph of the included studies. **Figure S3.** Baujat plot to assess the influence of the studies and it’s heterogeneity on the pooled estimates of sensitivity and specificity. **Figure S4.** Leave-one-out analysis plot assessing the influence of individual studies on the combined estimates of sensitivity and specificity. **Table S1.** Keywords and MeSH terms

**Supplementary Figure 1: Sensitivity Funnel Graph of the included studies**

**
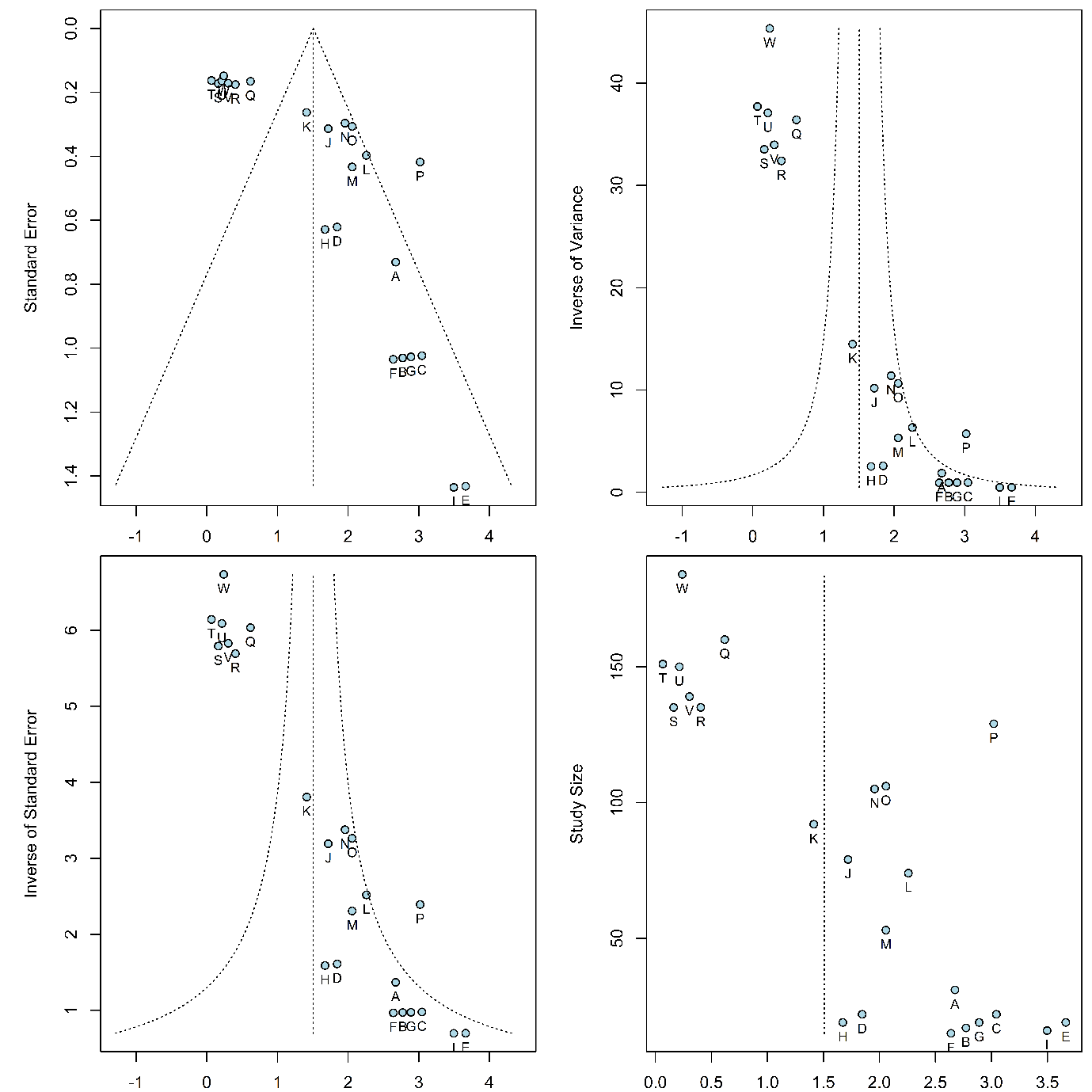
**

**Supplementary Figure 2: Specificity Funnel Graph of the included studies**

**
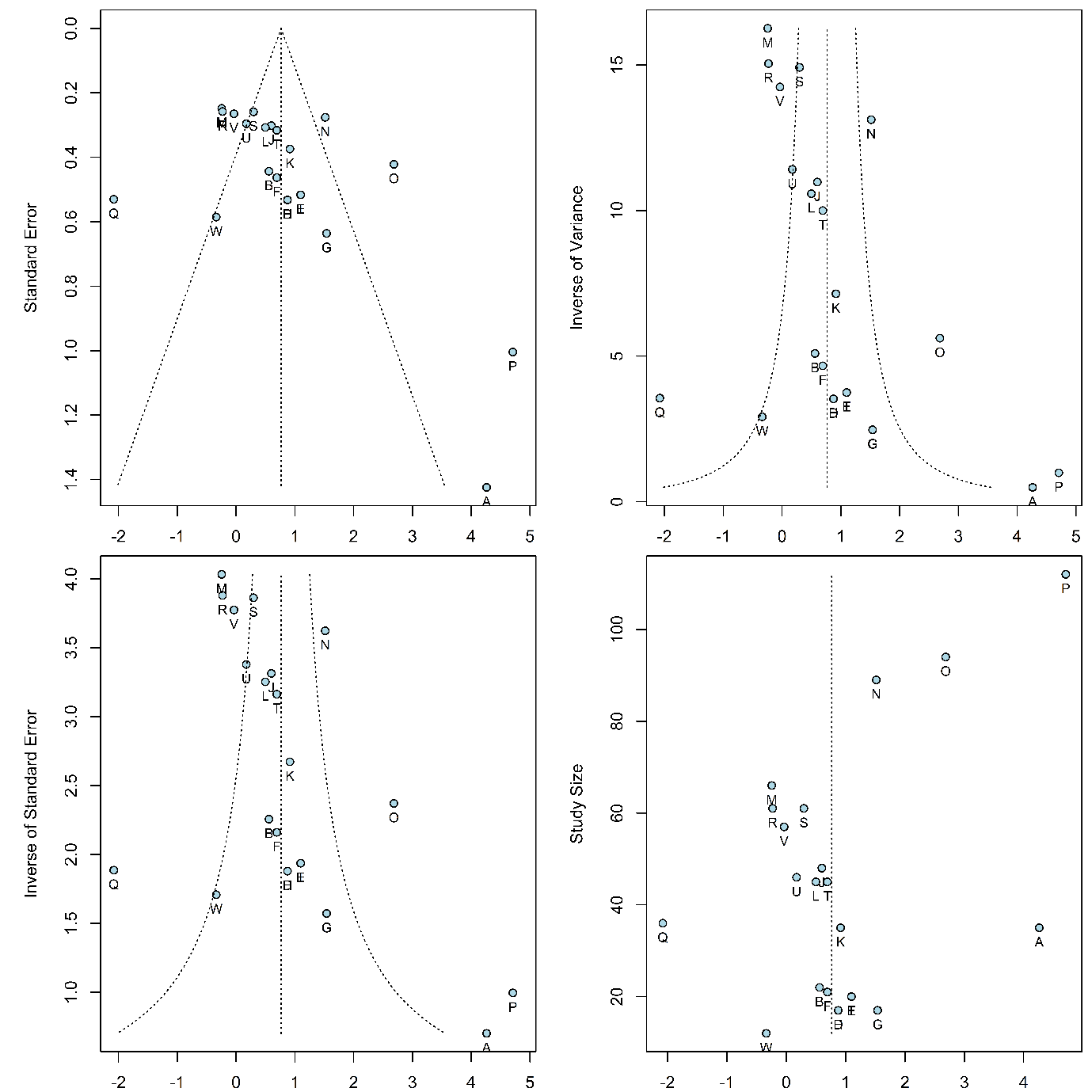
**

**Supplementary Figure 3: Baujat plot to assess the influence of the studies and it’s heterogenity on the pooled estimates of sensitivity and specificity**


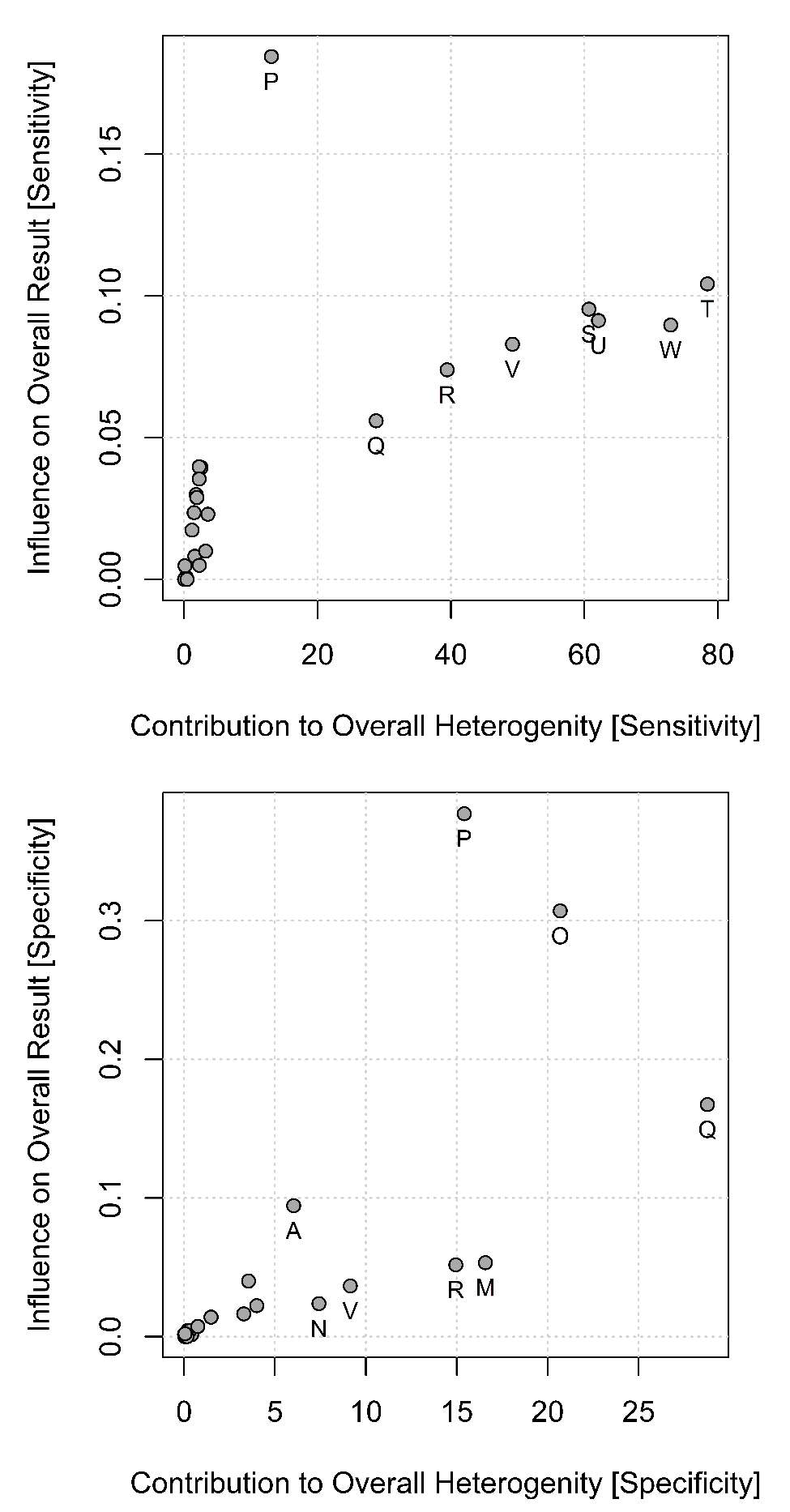


**Supplementary Figure 4: Leave-one-out analysis plot assessing the influence of individual studies on the combined estimates of sensitivity and specificity**

**
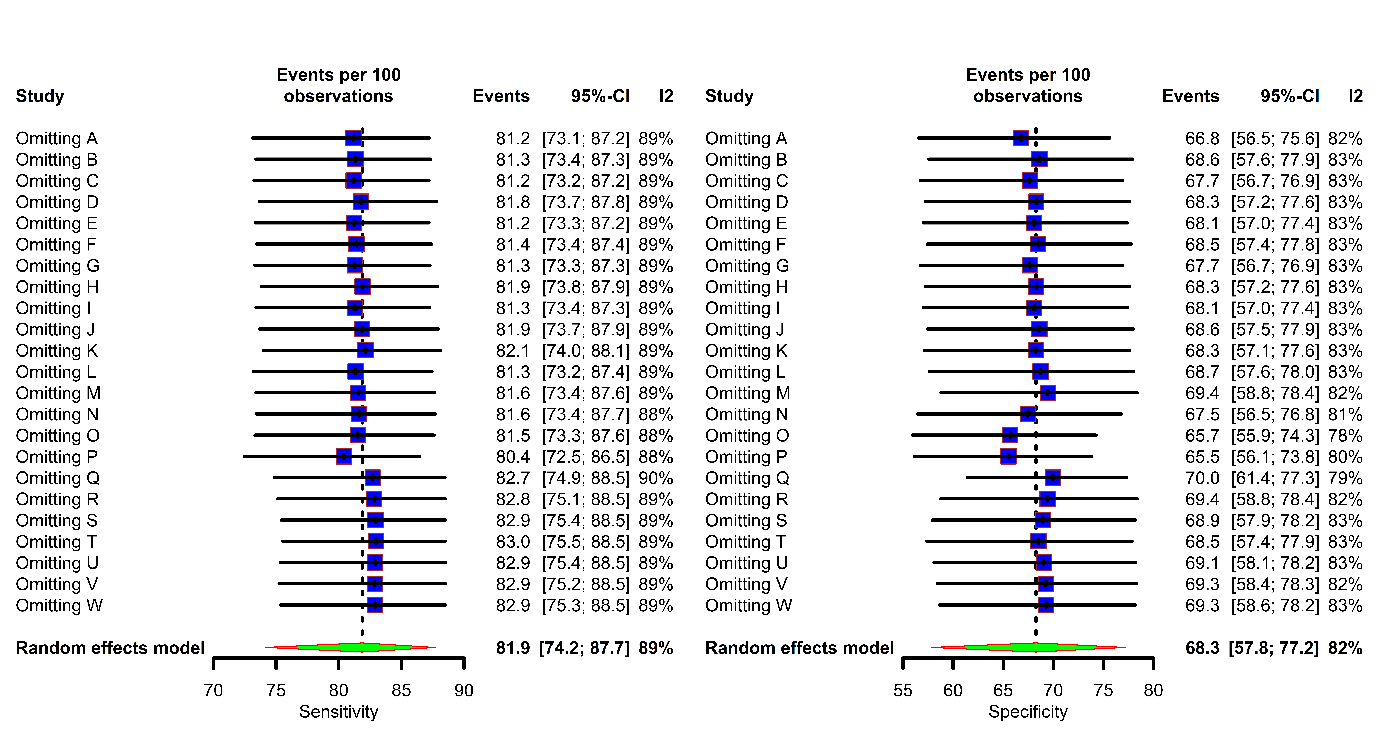
**

**Supplementary Table 1: Keywords and MeSH terms**

| S.No | Database | Search Year | Search Field | Search Terms | Other filters | Results | Exclusion | Inclusion |
| --- | --- | --- | --- | --- | --- | --- | --- | --- |
| 1 | Scopus | 2012-2021 | Title | miRNAs OR microRNAs AND  serum OR plasma OR circulation AND Tuberculosis | - | 13 | 2 | 11 |
| 2 | EMBASE | 2012-2021 | Article Title | Serum miRNAs OR serum microRNAs OR plasma miRNAs OR plasma microRNAs OR circulating miRNAs OR circulating microRNAs OR circulation miRNAs OR circulation microRNAs AND Tuberculosis | Quick limits: Human  English  Pub Type: Articles  Sources:  EMBASE, MEDLINE | 11 | 9 | 2 |
| 3 | Pubmed | 2012-2021 | MESH | Serum miRNAs OR serum microRNAs OR plasma miRNAs OR plasma microRNAs OR circulating miRNAs OR circulating microRNAs OR circulation miRNAs OR circulation microRNAs AND Tuberculosis | - | 90 | 70 | 20 |
| 4 | Cochrane |  | Record Title | Serum miRNAs OR Plasma miRNAs OR circulating miRNAs AND tuberculosis | - | 16 | 16 | 0 |
| 5 | Google Scholar | 2012-2021 |  | With all words: miRNAs Tuberculosis  With any one of the words: Serum Plasma circulation circulating | - | 5 | 5 | 0 |
| 6 | Web of Science | 2012-01-01 to 2021-12-31 |  | Tuberculosis AND Serum miRNAs OR serum microRNAs OR plasma miRNAs OR plasma microRNAs OR circulating miRNAs OR circulating microRNAs OR circulation miRNAs OR circulation microRNAs | Excluded: Review articles  Document Type: Article | 741 | 741 | 0 |
